# Analysis of annexin-A1 in the macrophages and apoptotic cells of patients with cutaneous leishmaniasis

**DOI:** 10.1590/0037-8682-0756-2020

**Published:** 2021-08-20

**Authors:** Mariana Nascimento Pona, Jordhana Mendonça Dietrich, Joselina Maria da Silva, Helen Aguiar Lemes da Silva, Marcia Hueb, Amilcar Sabino Damazo

**Affiliations:** 1 Universidade Federal de Mato Grosso, Faculdade de Medicina, Cuiabá, MT, Brasil.; 2 Universidade Federal de Mato Grosso, Programa de Pós-Graduação em Ciências da Saúde, Cuiabá, MT, Brasil.; 3 Universidade Federal de Mato Grosso, Faculdade de Medicina, Departamento de Ciências Básicas em Saúde, Cuiabá, MT, Brasil.; 4 Universidade Federal de Mato Grosso, Faculdade de Medicina, Departamento de Clínica Médica, Cuiabá, MT, Brasil.

**Keywords:** Cutaneous leishmaniasis, Skin, Macrophage, Apoptosis, Annexin-A1

## Abstract

**INTRODUCTION:**

This study aimed to determine the number of macrophages and apoptotic cells and perform annexin-A1 detection in patients with leishmaniasis.

**METHODS:**

Patients with *Leishmania* infection were admitted to Júlio Müller University Hospital.

**RESULTS:**

The number of apoptotic cells was higher in the exudative granulomatous reaction. The exudative cellular reaction displayed higher levels of annexin-A1 detection in macrophages and apoptotic cells. The correlation between annexin-A1 detection in apoptotic cells and macrophages was observed in exudative necrotic reaction and exudative necrotic-granulomatous reaction.

**CONCLUSIONS:**

Our data demonstrate the relevance of annexin-A1 in the regulation of apoptosis and phagocytosis in leishmaniasis.

American tegumentary leishmaniasis (ATL) is a non-contagious infectious disease caused by different species of the genus *Leishmania.* Transmission occurs through the bite of infected female phlebotomus, belonging to the genus *Lutzomyia* in the New World^1^. ATL may occur with diverse clinical manifestations, ranging from cutaneous lesions that are self-limited and regressive to multiple injuries and difficult to control^2^. The clinical presentation depends on the ability to control infection through the balance of cellular immune response^3^. Cutaneous leishmaniasis (CL) causes a vigorous inflammatory reaction, typically leading to skin ulceration within a few weeks, followed by a chronic skin ulcer that lasts for up to a year. *Leishmania* uses mechanisms such as apoptosis to escape from the harmful action of the host’s immune system^4^ ^,^ ^5^. The importance of apoptosis in modulating the immune response in humans with CL remains uncertain. However, some studies have indicated that the parasite may induce apoptosis of lymphoid cells, thereby affecting the profile of the cytokines produced and the regulation of their survival in the host. Apoptosis regulation is mediated by the involvement of several distinct receptor pathways and mediators. Among them, the protein annexin A1 (ANXA1) can be emphasized as a potent endogenous modulator of inflammation^3^ ^,^ ^6^ ^,^ ^7^, leukocyte apoptosis^8^, and apoptotic cell clearance^9^. Although several studies have evaluated ANXA1 actions, only few have focused on the CL-related infectious processes^4^ ^,^ ^10^. The present study aimed to determine the number of macrophages and apoptotic cells in the skin lesions of patients with leishmaniasis and analyze ANXA1 detection in these cells.

Patients with CL (n = 100) had a mean age of 46 years (range, 18-80 years). All patients were assisted at Leishmaniosis Outpatient Clinic, Julio Muller University Hospital (HUJM), at the Federal University of Mato Grosso (UFMT), Cuiabá-MT, Brazil. Patients with CL who had no chronic degenerative or infectious immunosuppressive diseases and had not started treatment were considered eligible for this study.

Participants signed the Free and Informed Consent Term as an agreement to participate in the study. This study was approved by the Research Ethics Committee HUJM (51430915.0.0000.55.41). All procedures were performed in accordance with the Declaration of Helsinki.

The lesion margin was cleaned, and 2% lidocaine was used for inducing local anesthesia. In biopsy, a 4-mm "punch" was performed. The skin fragments were fixed in 4% paraformaldehyde/phosphate-buffered saline (PBS) solution and transported to the Laboratory of Histology at the medical school. The biopsy specimen was washed in PBS, dehydrated in ethanol, diafanised in xylene, and embedded in paraffin.

Skin samples were cut at 3-μm thickness using a HIRAX M60 microtome (Carl Zeiss, GR). The sections were placed on histological slides, dewaxed, rehydrated, and stained with hematoxylin-eosin for histopathological analysis. Two blinded microscopists classified the lesions depending on the histopathological characteristics: exudative cellular reaction (ECR), exudative tuberculoid reaction (ETR), exudative granulomatous reaction (EGR), exudative necrotic reaction (ENR), and exudative necrotic-granulomatous reaction (ENGR). This classification was in accordance with that of Magalhães and collaborators^11^.

*Leishmania* was identified by polymerase chain reaction (PCR), wherein the gene HSP70C was targeted, and it was performed as described previously^10^. Positive HSP70C PCR products were then used in the restriction fragment length polymorphism (RFLP) technique, using the *Hae*III and *BstU*I enzymes as described previously.

For endogenous ANXA1 detection in apoptotic cells and macrophages, 3-μm-thick skin sections were placed on slides with BIOBOND (British Biocell International, Cardiff, UK), a biological adhesive. This technique was performed as described previously^3^. The slides were incubated in a water bath at 70°C in 0.21% sodium citrate solution for 1 h; they were then blocked with 3% hydrogen peroxide in 70% methanol for 1 h, followed by permeabilization by incubation with 0.4% Tween 20 in PBS for 15 min. The slides were blocked again with 5% bovine serum albumin (BSA), diluted in PBS, for 1 h and were incubated with primary antibodies overnight at 4°C in a humid tray. To detect ANXA1, we used rabbit anti-ANXA1 (1:200, 1% BSA; Invitrogen, USA); to identify macrophages, we used anti-CD163 mouse (1:200; Cell Marque, USA, clone EP152); to detect cleaved caspase-3, we used mouse monoclonal cleaved anti-caspase-3 (1:200; Cell Signaling, USA). The secondary antibodies used were as follows: Alexa Fluor 488 fluorochrome-conjugated goat anti-rabbit IgG (1:200 in 1% BSA; Invitrogen, USA), Alexa Fluor 555 fluorochrome-conjugated goat anti-mouse IgG (1:200; Invitrogen, USA), and DAPI (4′,6-diamidino-2-phenylindole) nucleus marker (Sigma, USA), incubated for 1 h at 27°C in the dark. The slides were washed with PBS and mounted on Citiflour (DAKO, USA). Immunolabeled macrophages and apoptotic cells were quantified using a AxioScope A1 microscope (Carl Zeiss, GR) using Axiovision software (Carl Zeiss, GR). Cells were quantified as mean ± standard deviation (SD)/mm^2^ for lesions in patients with CL. ANXA1 detection was measured by average optical density, using Axiovision software, using the light spectrum [value arbitrarily assigned, ranging from 0 to 255 arbitrary units (AU)]. ANXA1 expression in macrophages and apoptotic cells was described as mean ± standard deviation (SD) in the CL lesion.

The results obtained were statistically evaluated using one-way analysis of variance with Bonferroni post-hoc test and with linear regression analysis. GraphPad Prism 5 software (La Jolla, CA, USA) was used. Statistical significance was set at p < 0.05.

PCR-RFLP analysis indicated that all CL patients were infected with *L. (Viannia) braziliensis*. Histopathological analysis included patients with each pathological type of lesion, previously classified as ECR (n=64), EGR (n=20), ENR (n=11), and ENGR (n=5). In this study, there were no patients with ETR lesions. In ECR patients, the skin lesions were ulcerated, hyperemic, and infiltrated. The mean time between symptom onset and diagnosis was 2 months. In patients with EGR, the skin lesions infiltrated and had a granular surface, with a mean time of 4 months. In patients with ENR, the skin lesions appeared ulcerated, with raised borders, and infiltrated, with a mean time of 3 months. In patients with ENR and ENRG, the skin lesions appeared ulcerated, with raised borders, and infiltrated, with a mean time of 3 months. The number of lymphocytes, plasma cells, and neutrophils were not significantly different between the histopathological types. Multinucleated giant cells were found in higher numbers in ENGR; kinetoplasts occurred in a lower number in ENR, and edema was identified in smaller quantities in EGR lesions (Table 1).

**TABLE 1: Cellular:** elements and edema by pathological type.

	Lymphocyte	Plasmocyte	Multinucleated giant cell	Kinetoplast	Neutrophil	Edema
**ECR**	2.1 ± 0.2	1.3±0.1	0.1±0.1	1.6±0.2	0.2±0.1	1.8±0.2
**EGR**	3.0 ± 0.2	1.4±0.2	0.4±0.1**	1.4±0.2	0.2±0.1	1.2±0.2*
**ENGR**	2.5 ± 0.2	1.2 ± 0.1	0.6±0.1***	1.2±0.2	0.3±0.1	2.2±0.2
**ENR**	1.6 ± 0.2	1.3 ± 0.2	0.07±0.1	0.9±0.2*	0.2±0.1	2.1±0.3

* p < 0.05; ** p < 0.001; *** p < 0.0001 when compared to ECR.

Data are presented as average and standard deviation. **ECR:** exudative cellular reaction; **EGR:** exudative granulomatous reaction; **ENGR:** exudative necrotic-granulomatous reaction; **ENR:** exudative necrotic reaction.

In the quantification of ANXA1, a higher concentration of this protein was observed in macrophages present in the lesions of the ECR type (88.7±1.6 AU), while EGR, ENR, and ENGR showed lower detection (52.2±1.4, 57.8±1.7, and 62.8±2.3, respectively) (Figure 1A-C).

Similarly, the apoptotic cells in patients with lesions of the ECR type expressed more ANXA1 (94.6±1.7) than the other histopathological types (Figure 1D-F).

Regarding the number of apoptotic cells by histopathological type, a lower number of cells was observed in ECR (74.0±12.8), whereas a higher number was found in EGR (191.0±8.4) (Figure 1G).

**FIGURE 1:**
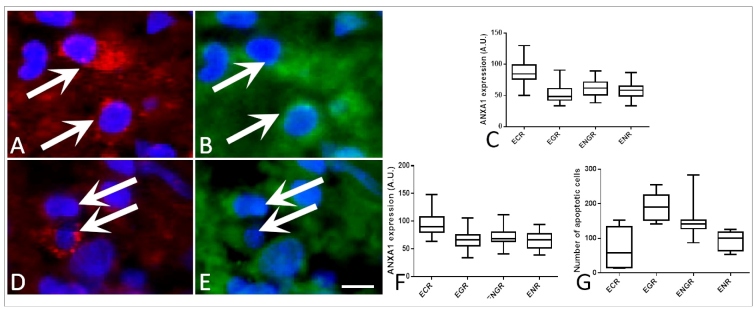
Analysis of annexin A1 detection in macrophages and apoptotic cells in the leishmaniasis lesion. **(A)** Identification of macrophages stained by the antibody CD163 (arrows). **(B)** Annexin A1 expression in the macrophage cytoplasm (arrows). **(C)** ANXA1 detection in the macrophages present in different types of leishmaniasis lesions. **(D)** Identification of apoptotic cells stained by cleaved caspase 3 (arrows). **(E)** Annexin A1 expression in the apoptotic cells (arrows). **(F)** ANXA1 detection in apoptitic cells present in different types of leishmaniasis lesions. **(G)** Number of apoptotic cells in the skin lesions of patients with leishmaniasis. Scale Bar = 50 μm.

Regression analysis showed that ENR (R^2^=0.4845) and ENGR (R^2^=0.4807) feature correlations in the detection of ANXA1 between macrophages and adjacent apoptotic cells, while ECR and EGR did not show such a correlation (Figure 2).

**FIGURE 2:**
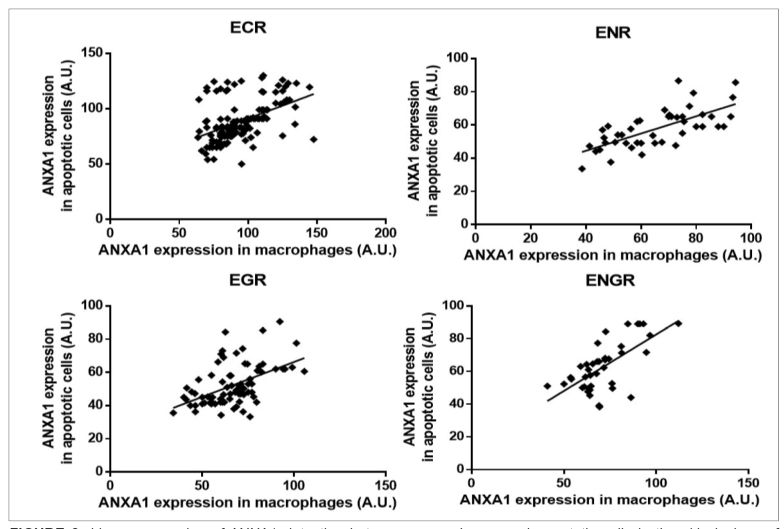
Linear regression of ANXA1 detection between macrophages and apoptotic cells in the skin lesions of patients with leishmaniasis. **ECR:** exudative cellular reaction; **EGR:** exudative granulomatous reaction; **ENGR:** exudative necrotic-granulomatous reaction; **ENR:** exudative necrotic reaction.

In this study, we observed that ANXA1 detection in macrophages and apoptotic cells varied depending on the type of histopathological site. The characteristics of the infectious process of CL depend on the presence of specific types of leukocytes and the release of immunological mediators at the inflammatory site^13^. ANXA1 has been shown to be a regulatory mediator of leukocyte activation and phagocytic processes during *Leishmania* infection^4^ ^,^ ^10^.

All patients were infected with *Leishmania (Viannia) braziliensis*. Similar data were reported by Silva and collaborators^3^, which were also observed in CL patients from Cuiabá, MT, Brazil, wherein the majority of the species was *L. braziliensis*. 

The macrophages in the ECR lesions expressed higher levels of ANXA1. This protein leads to the activation of non-phlogistic phagocytosis^9^ and controls the release of cytokines^6^ ^-^ ^8^. According to the study by de Magalhães and collaborators^11^ on the evolution of CL injuries, ECR is the initial framework of the disease, characterized by a nonspecific chronic inflammatory process, in which the mechanism of self-control is in progress. In addition, the same author found an inversely proportional relationship between the time of infection and the number of parasites. Apoptotic cells were present in lower numbers in the ECR lesions than in the other groups. However, ANXA1 expression was higher in these lesion types. This protein can mediate pro-apoptotic effects by activating caspase-3 and by acting on the influx of intracellular calcium^8^. Thus, ANXA1 detection appeared to be increased in both apoptotic cells and macrophages. This facilitates the induction of apoptosis and the clearance of apoptotic cells by macrophages^9^. It is important to highlight that the induction of apoptosis in CL lesions can be due to unsuccessful attempts at phagocytosis and destruction of parasites. Non-phlogistic phagocytosis by macrophages might contribute to cell inoculation by *Leishmania* through a Trojan horse mechanism^12^.

A converse situation was observed in patients with ENGR and EGR lesions, wherein macrophages and apoptotic cells present a lower expression level of ANXA1 and there are a higher number of apoptotic cells. Both lesions have granulomas, a structure with an isolated microenvironment that tends to generate a lower phagocytic activity and an intense production of cytotoxic cytokines such as interferon-γ and tumor necrosis factor-α^14^. Therefore, ANXA1 would be less expressed, potentiating the release of these cytokines.

By performing a linear regression analysis between the detection of ANXA1 in macrophages and apoptotic cells, it was found that the lesions of the ERN and ENGR types presented a significant correlation. This could indicate specific activation of macrophages outside tissue necrosis. Previous studies have demonstrated that the ENGR type develops a post-necrotic granulomatous reaction^15^. Therefore, ENR and ENGR feature a similar extent of tissue activation, maintaining the detection of ANXA1 between macrophages and apoptotic cells.

In conclusion, the detection of ANXA1 in macrophages present in ECR lesions is related to the non-phlogistic phagocytic activity. Higher expression levels of ANXA1 occur at the induction of the apoptotic mechanism in the ECR lesions, which induces the clearance of these cells by phagocytosis. The action of ANXA1 in the induction of apoptosis and non-phlogistic phagocytosis in ECR lesions might be related to the propagation of *Leishmania* through the Trojan horse mechanism.
